# Letter to the Editor: Balancing the Pendulum-The Risks of Universal Fluid Restriction and Over-reliance on “Static” Phase Models

**DOI:** 10.1016/j.aicoj.2026.100187

**Published:** 2026-10-16

**Authors:** Tanmoy Ghatak, Kalavathy Swarna

**Affiliations:** a Sanjay Gandhi Postgraduate Institute of Medical Sciences, India; b SIMS Hospital Vadapalani, Chennai, India

We read with great interest the review by Vanden Eede and Van Regenmortel on a phase-adapted approach to fluid therapy in shock [1]. The authors carefully caution that fluid restriction should not be applied to non-resuscitated patients in the initial phase and that management must be individualised, while noting an overall trend towards restriction across the ICU stay given the association between fluid overload and mortality. We fully endorse this individualised ‘patient-level titration’ philosophy. Our concern is with the potential risk that a broadly framed “restrictive” or “de-resuscitation” could become a new source of iatrogenic harm if we are not careful.

The review advocates restrictive strategies principally to reduce acute kidney injury and acute respiratory distress syndrome (ARDS), and the established association between fluid overload and mortality supports caution about cumulative balance. A recent meta-analysis found that restrictive strategies reduce the duration of mechanical ventilation but do not significantly reduce all-cause mortality compared with liberal strategies (risk ratio 0.99; 95% CI, 0.90–1.08) [2]. This neutral mortality signal, however, is difficult to interpret, as many included trials enrolled patients only after they had already received large volumes of fluid before randomisation, and thus could not properly test a restrictive strategy. Moreover, a restrictive philosophy applies chiefly to maintenance, replacement and stabilisation fluids and does not imply inadequate resuscitation during the “Rescue” phase, in which the review itself recommends small, repeated boluses (4 mL/kg or 250–500 mL) with individualised assessment. Early, adequate volume remains the cornerstone of surviving sepsis.

The review presents the ROSE (Resuscitation, Optimisation, Stabilisation, Evacuation) model as a conceptual framework encouraging contextual interpretation rather than a rigid bedside algorithm. Even so, operationalising its phase boundaries is difficult in practice: fluid accumulation trajectories often lack distinct “transition points”, making the Stabilisation phase hard to identify in real time [3]. Without validated, objective triggers for deescalation, clinicians may start diuretics prematurely, potentially compromising organ perfusion in patients who have not yet reached true haemodynamic stability. Objective triggers for de-escalation therefore remain an unresolved but pertinent question. Reassuringly, emerging data suggest that diuretics may be used safely even early and in patients still receiving vasopressors to limit fluid balance [4,5].

The article suggests starting vasopressors early to minimise fluid volumes. The review explicitly states that decisions should be individualised to balance volume expansion with restoration of vascular tone, and we agree. The evidence base is genuinely mixed: a recent meta-analysis reported a mortality benefit with early norepinephrine [6], whereas in patients with preserved diastolic blood pressure (DBP ≥50 mmHg) early norepinephrine administration showed no mortality benefit [7]. The central difficulty is that “early” is defined heterogeneously across studies – for example, approximately 25 min within a 3 -h window in the latter cohort, which might be considered late elsewhere. We therefore argue not against early vasopressors but for a clearer, phenotype-aware definition of timing [8].

The review presents the Venous Excess Ultrasound (VExUS) score not as a universal requirement but as one component of a broader, multimodal assessment of fluid tolerance. Our reservation concerns its inter-operator variability and therefore reproducibility, together with recognised limitations in patients with tricuspid regurgitation or atrial fibrillation – conditions that are common in the elderly septic population [9]. Congestion assessment should therefore be multimodal, and readily available indices such as clinical examination, cumulative fluid balance and central venous pressure continue to retain a useful complementary role [10].

## Conclusion

Reducing “fluid creep” is essential, but we must ensure that the move towards restriction does not become a dogmatic retreat from necessary resuscitation. A truly personalised approach needs to account for different haemodynamic phenotypes and the logistical realities of the bedside clinician.

## Funding

No funding from anywhere any institution or government.

## Prior abstract publication/presentation

No such presentation in any conference.

## Declaration of competing interest

There is no conflict of interest of any authors involved in this manuscript.
